# Beyond immunomodulation: The regenerative role for regulatory T cells in central nervous system remyelination

**DOI:** 10.1007/s12079-017-0392-8

**Published:** 2017-05-11

**Authors:** Veronique E. Miron

**Affiliations:** Medical Research Centre for Reproductive Health, The Queen’s Medical Research Institute, The University of Edinburgh, 47 Little France Crescent, Edinburgh, Scotland EH16 4TJ UK

**Keywords:** Remyelination, Oligodendrocyte, Multiple sclerosis, Regulatory T lymphocyte, CCN3, Regeneration, Myelin

## Abstract

Central nervous system regeneration after injury can occur in the form of remyelination, the reinstatement of myelin around axons which restores axon health and function. However, remyelination often fails in chronic neurological diseases, such as progressive multiple sclerosis. The lack of currently approved pro-remyelination therapies highlights the need to elucidate the cellular and molecular mechanisms underpinning this regenerative process. Whereas some T lymphocyte subsets such as Th1 and Th17 are implicated in inducing myelin injury, a recent study by Dombrowski et al. reveals a novel role for regulatory T cells (T_regs_) in directly driving remyelination, independent of immunomodulation (Nat Neurosci doi:10.1038/nn.4528 2017)(Dombrowski et al., 2017). This study is summarized in this Bits and Bytes.

Regeneration can occur efficiently in the central nervous system (CNS) in the form of remyelination, whereby new myelin is ensheathed around axons to reinstate trophic/ metabolic support and insulation for electrical impulse conduction. This process requires oligodendrocyte progenitor cells (OPCs) to migrate to areas of injury, proliferate, and differentiate into myelin-producing oligodendrocytes. Remyelination is limited or fails altogether in various neurological disorders, most prominently in progressive multiple sclerosis (MS), leading to axon dysfunction or loss. Although it is recognized that this failure largely reflects impaired oligodendrocyte differentiation (Kuhlmann et al., 2008), the mechanisms underpinning successful remyelination are still not fully understood. This highlights the importance of identifying the cells and molecules driving remyelination in order to develop effective regenerative therapies.

While it is now recognized that the innate immune system (i.e. macrophages) supports remyelination (Davies and Miron, 2016; Lloyd and Miron, 2016), the adaptive immune system (e.g. T lymphocytes) has historically been considered deleterious for oligodendrocytes/ remyelination. For instance, pro-inflammatory Th1 and Th17 T cells have direct cytotoxic effects on human OPCs in vitro (Moore et al., 2015) and reduce remyelination in vivo (Baxi et al., 2015). However, the impairment of remyelination following depletion of total CD4+ or CD8+ T cell populations (Bieber et al., 2003) points to the existence of a pro-regenerative T cell subset. Consistent with this postulate, a T cell presence in MS lesions is concurrent with ongoing remyelination. Indeed, a recent study by Dombrowski and Fitzgerald and colleagues (Dombrowski et al., 2017) revealed a novel pro-regenerative role for regulatory T cells (T_regs_), demonstrating that these cells directly stimulate remyelination independent of immunomodulation.

Using a focal model of toxin-induced demyelination in the mouse spinal cord, where the temporal distinction between myelin damage and remyelination allows investigations of the regenerative process in isolation, T_regs_ (CD3+ CD4+ Foxp3+) were detected in lesions at the time of oligodendrocyte differentiation and remyelination initiation (Dombrowski et al., 2017). These cells were found to be required for remyelination, as their specific depletion in a Foxp3-driven diphtheria toxin receptor model (Foxp3-DTR) led to reduced numbers of oligodendrocytes and remyelinated axons (Dombrowski et al., 2017). This was rescued by supplementation with exogenous wildtype T_regs_. The effects of T_reg_ depletion were mirrored in a distinct demyelination model (Dombrowski et al., 2017), whereby select oligodendrocytes in the brain are killed via copper chelation by the cuprizone toxin, confirming that the role of T_regs_ in remyelination is not dependent on the mode of demyelination nor is it restricted to the spinal cord.

T_reg_ depletion in both models did not alter numbers of total oligodendrocyte lineage cells nor proliferating OPCs, suggesting effects of T_regs_ on the differentiation of OPCs into mature myelinating oligodendrocytes. Indeed, exposing brain explants to T_regs_ or their conditioned media enhanced oligodendrocyte differentiation, myelination, and remyelination, in comparison to non-polarized CD4+ T cells (Dombrowski et al., 2017). These effects were independent of immunomodulation of i) the peripheral immune system, as explants are devoid of a circulation, and ii) microglia and astrocytes, as effects were still observed when their inflammatory response to dissection had subsided (Dombrowski et al., 2017). A direct effect of T_regs_ on OPCs was confirmed in vitro, where T_reg_ conditioned media enhanced the differentiation of isolated OPCs and accelerated myelination in OPC-neuronal co-cultures (Dombrowski et al., 2017). Proteomic profiling of T_reg_ conditioned media was carried out to identify pro-remyelination factors, and identified high expression of the growth regulator CCN3 (Dombrowski et al., 2017). This factor has previously been shown to be involved in tooth regeneration (Wang et al., 2014), but never shown to be expressed either by T cells or during CNS regeneration. CCN3 was found to be a critical component of the beneficial effects of T_reg_ conditioned media, as use of a blocking antibody or specific depletion from the conditioned media abolished the pro-differentiation and pro-myelination effects (Dombrowski et al., 2017). Importantly, T_reg_-derived CCN3 was sufficient to support these responses (Dombrowski et al., 2017).

Altogether, these data demonstrate that T_regs_ are direct drivers of oligodendrocyte differentiation and remyelination, thereby revealing a novel regenerative function for T_regs_ beyond immunomodulation. Considering this study, one must now acknowledge that the adaptive immune system is not solely involved in damage induction and modulation of inflammation, but is also a critical component of the regenerative process that follows. A recent study demonstrated that MS patient-derived CD4+ T cells injected into demyelinated mouse CNS show high variability in their ability to support remyelination (El Behi et al., 2017), leading to the tantalizing possibility that the inter-patient variation in remyelination efficiency in MS might reflect diverging capacity of T_regs_ to stimulate remyelination. Overall, these studies support that further investigations into the pro-remyelination function of T_regs_ and CCN3 in MS are warranted for development of novel regenerative therapies.
